# Synergic Transformation of an Ethylenediamine to a Lithium 1,3-Diaza-2-zincacyclopentene via an Alkyllithium/Bis(alkyl)zinc Mixture

**DOI:** 10.1002/chem.201001314

**Published:** 2010-07-19

**Authors:** Ross Campbell, Pablo García-Álvarez, Alan R Kennedy, Robert E Mulvey

**Affiliations:** [a] WestCHEM, Department of Pure and Applied Chemistry, University of StrathclydeGlasgow G1 1XL (UK)

**Keywords:** amines, hydrides, lithium, metallacycles, zinc

Alkali metal zincates are among an increasing number of mixed-metal organoreagents that are attracting widespread attention because of their ability to exhibit synergic reactivity. Such special behaviour can be defined as reactions arising from the cooperative effects of the two distinct metals, the hard alkali metal and soft zinc, within the multicomponent zincate that cannot be reproduced by either the single alkali metal or zinc component on its own. This synergism has been particularly prominent in metallation (metal-hydrogen exchange) applications.^1^ Alkylzinc (R_2_Zn) or amidozinc [RZn(NR′_2_)/Zn(NR′_2_)_2_] reagents are generally notoriously poor kinetic bases incapable of directly metallating (zincating) aromatic substrates to any synthetically useful extent, but combined with an alkali metal compound^2^ or related component^3^ they can transform into highly reactive “zincators”. Kondo and Uchiyama’s “LiZn(TMP)*t*Bu_2_” (TMP = 2,2,6,6-tetramethylpiperidide), Mongin’s “LiZn(TMP)_3_”,^[^^2a^^–^^j^^]^ and our own [(TMEDA)Na(TMP)(*t*Bu)Zn(*t*Bu)]^[^^2p^^–^^t^^]^ (TMEDA = *N*,*N*,*N′*,*N′*-tetramethylethylenediamine) belong in this category and although fundamental differences exist between these powerful amide-based zincators, their Zn-H exchange reactions can be grouped together as alkali-metal-mediated zincations “AMM*Z*n’s”.^1^ Exemplified by TMP, the amide components involved in AMM*Z*n chemistry are invariably monoanionic ligands derived from secondary amines containing one acidic N–H bond. Secondary diamines containing two acidic N–H bonds and therefore having potentially at least two metallation sites would offer an interesting contrast but until this work they have not been investigated in this context. Reported herein, our first venture in introducing diamines to this chemistry by reacting a mixture of *n*BuLi and *t*Bu_2_Zn (or Me_2_Zn) with *N*,*N*′-diisopropylethylenediamine, *i*PrN(H)CH_2_CH_2_N(H)*i*Pr, has uncovered a surprising new synergic chemistry, which leads ultimately to the transformation of the saturated ethylenediamine to a dianionic unsaturated diazaethene.

To gauge whether a bimetallic mixture exhibits synergic activity, its separated monometallic components should be reacted with the substrate in control reactions. As expected, we found that *t*Bu_2_Zn is too weak a base to deprotonate *N*,*N′*-diisopropylethylenediamine, but instead forms the simple Lewis acid-Lewis base adduct [*t*Bu_2_Zn**·** {*i*PrN(H)CH_2_CH_2_N(H)*i*Pr}] (**1**; Scheme 1: see the Supporting Information for full details). While we easily generated a crystalline product from a 1:1 reaction of the more powerful base *n*BuLi and the diamine, and characterised it spectroscopically as [{LiN(*i*Pr)CH_2_CH_2_N(H)*i*Pr}], we deemed it unnecessary to determine its crystal structure as Gardiner and Raston had previously reported^4^ that the same reaction with the *tert*-butyl homologue *t*BuN(H)CH_2_CH_2_N(H)*t*Bu produced lithiation of one N–H unit in [*cis*-{Li[μ-N(*t*Bu)CH_2_CH_2_N(H)*t*Bu]}_2_] (**2**), which is dimeric with a 5,4,5-fused ring system having a (LiN)_2_ core (Scheme 1). From the precedents of these homometallic zinc and lithium control reactions, in the absence of any co-operative interactions between the distinct metals one might anticipate that reaction of a 1:1 mixture of *t*Bu_2_Zn and *n*BuLi with *N*,*N*′-diisopropylethylenediamine in the presence of TMEDA (one molar equivalent) would produce a co-complex of composition [*t*Bu_2_Zn{*i*PrN(Li⋅TMEDA)CH_2_CH_2_N(H)*i*Pr}] (**3**). Kinetically it does and we obtained the crystal structure of **3** (Figure 1). However, thermodynamically, this reaction mixture in hexane solution surprisingly affords the crystalline product [(TMEDA)Li(*i*PrNCHCHN*i*Pr)Zn(*t*Bu)] (**4**). As depicted in Scheme 1, formation of this lithium 1,3-diaza-2-zincacyclopentene formally requires the loss of two protons and hydrogen gas to transform the neutral saturated ethylenediamine to a dianionic unsaturated variant. To check whether this transformation was the result of employing two or more equivalents of the metal reagent and/or adding TMEDA, a 3:1:0 and 3:1:3 mixture of *n*BuLi, diamine, and TMEDA, in the absence of *t*Bu_2_Zn, was evaluated but NMR spectroscopic studies confirmed only synthetically insignificant trace amounts of a diazaethene product with the major product being an ethylenediamine complex in which the -NCH_2_CH_2_N- bridge is retained.^5^ On this evidence, the double (sp^3^) C-H bond activation and concomitant C=C formation involved in the making of the metallocycloalkene **4** can be attributed at least in part to a special bimetallic synergic effect under the particular conditions studied, though other factors such as changing concentration may also be important as C=C bond formation has been observed to occur in concentrated monometallic systems (see below).

**Scheme 1:**
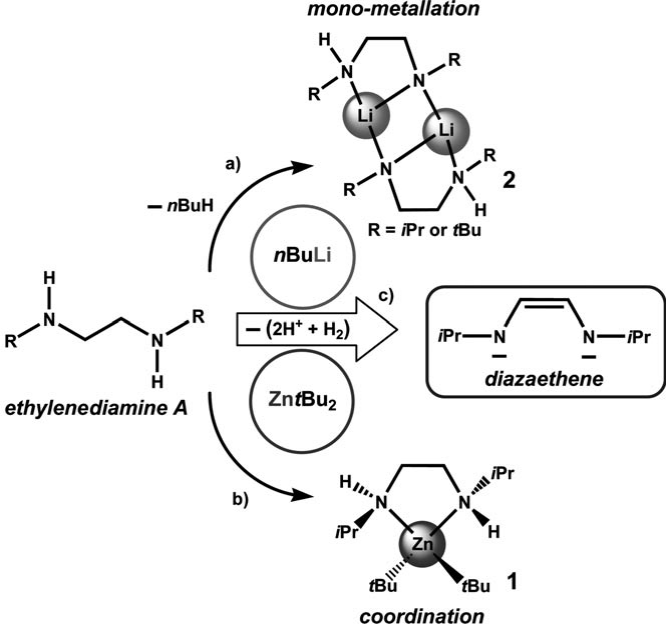
Comparison of non-cooperative reactions [*n*BuLi (a) or *t*Bu_2_Zn (b)] and cooperative reactions [*n*BuLi and *t*Bu_2_Zn (c)] of metal alkyls with an ethylenediamine A. Reaction conditions: a) hexane at 25 °C, 1 h; b) hexane at 25 °C, 24 h and at 69 °C, 10 min; and c) hexane at 69 °C, 2 h.

**Figure 1:**
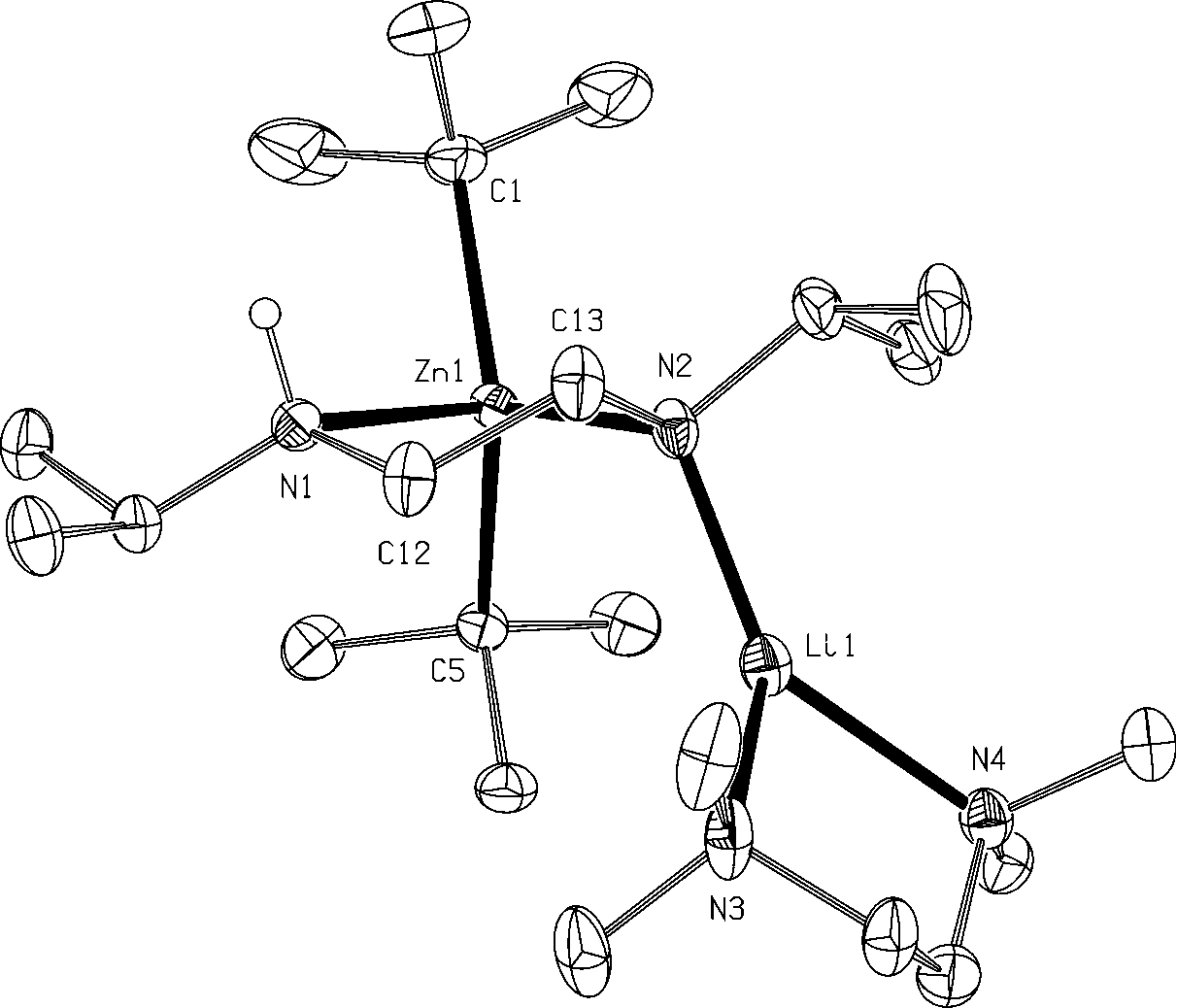
Molecular structure of 3 with hydrogen atoms (except N*-*H) omitted for clarity. Selected bond lengths [Å] and angles [^o^]: Zn1-C1 2.073(2), Zn1-C5 2.068(2), Zn1-N1 2.326(1), Zn1-N2 2.127(1), Li1-N2 1.993(3), Li1-N3 2.143(3), Li1-N4 2.173(3), N1-C12 1.470(2), N2-C13 1.461(2), C12-C13 1.517(2); C5-Zn1-C1 125.65(7), C5-Zn1-N2 111.49(6), C5-Zn1-N1 108.98(5), C5-Zn1-N2 111.49(56), C1-Zn1-N1 102.67(6), N2-Zn1-N1 83.10(5), N2-Li1-N3 127.12(15), N2-Li1-N4 141.1(2), N3-Li1-N4 87.1(1).

Determined by X-ray crystallography, the molecular structures of **3** (Figure 1) and **4** can be classed as contact ion pair zincates comprising a TMEDA-chelated lithium cation and an alkyl(diamido)zinc anion. Within **3** distorted tetrahedral Zn1 completes a highly puckered C_2_N_2_Zn metallacyclic ring (see, for instance, the torsion angle Zn1-N2-C13-C12 = −48.82(17)^o^) with an N-Zn-N bite angle of 83.10(5)° with *exo t*Bu substituents on Zn and *i*Pr/H and *i*Pr/Li substituents on N1 and N2, respectively. The *i*Pr groups both occupy equatorial sites and lie *anti* to each other across the five-atom ring which exhibits an ethylene C12-C13 bond length of 1.517(2) Å consistent with a single bond. Trigonal-planar Li1 has an N_3_ coordination comprising one diamine atom and two N atoms of TMEDA with the formal anionic Li-N2 bond (1.993(3) Å) being shorter than the latter dative Li-N bonds (mean length, 2.158 Å). Though the connectivity within **4** can be crystallographically determined, generic twinning of the samples adversely effected all modelling attempts and rules out discussion of its bonding dimensions. However, using an identical procedure to that for **4**, we synthesised and crystallographically characterised the isostructural methyl homologue [(TMEDA)Li(*i*PrNCHCHN*i*Pr)Zn(Me)] (**5**), the crystal data for which are more accurate allowing such a discussion. In the molecular structure of **5** (Figure 2) the anionic moiety shows N,N′-chelation by the N-C=C-N unit to zinc (bite angle, 83.98(9)°) to build a five-atom metallacycle with *exo i*Pr and Me substituents on N and Zn atoms, respectively. Distorted trigonal planar zinc deviates modestly from the C_2_N_2_ plane, bonding symmetrically to the N atoms (lengths, 1.966(2) and 1.964(2) Å; Zn 0.459(3)Å out of plane). In a typical range for sp^2^–sp^2^ C=C bonds, the C5-C6 bond length is 1.349(3) Å. The Li^+^ ion of the cationic moiety π bonds (η^4^-) asymmetrically to the N-C=C-N unit, closer to one end (Li1-N1, 2.165(4), Li1-C5, 2.235(4) Å) than the other (Li1-N2, 2.301(4); Li1-C6, 2.286(4) Å). This asymmetry continues with Li *anti* to Zn, with respect to the C_2_N_2_ plane, and separated from it by 2.688(4) Å. Though new for Li:Zn combinations, the alkali metal face-capped 1,3-diaza-2-metallocyclopentene motif of **4** and **5** is known for other combinations including for example Na:Zn,^6^ K:Zn,^7^ K:Mg,^8^ Li:Ga,^9^ or K:Ga.^10^ Most of these structural precedents originate from elemental alkali metal reduction of 1,4-diaza-1,3-butadienes or related unsaturated molecules which sets them synthetically apart from **4** and **5** as to the best of our knowledge these two Li:Zn examples represent the first to be synthesised from a completely non-activated aliphatic diamine.

**Figure 2:**
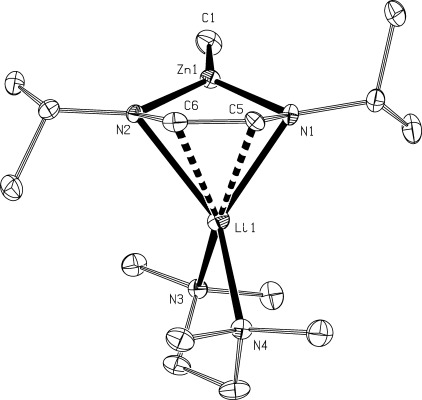
Molecular structure of 5 with hydrogen atoms omitted for clarity. Selected bond lengths [Å] and angles [^o^]: Zn1-C1 1.956(3), Zn1-N1 1.966(2), Zn1-N2 1.964(2), Zn1-Li1 2.688(4), Li1-N1 2.165(4), Li1-N2 2.301(4), Li1-N3 2.145(4), Li1-N4 2.106(4), Li1-C5 2.235(4), Li1-C6 2.286(4), N1-C5 1.398(3), N2-C6 1.402(3), C5-C6 1.349(3), N1-C5 1.398(3); C1-Zn1-N1 136.6(1), C1-Zn1-N2 138.69(9), N2-Zn1-N1 83.98(9), N2-Li1-N3 112.5(2), N4-Li1-N3 86.51(16), N4-Li1-N1 126.8(2), N3-Li1-N1 124.6(2), N4-Li1-N2 139.9(2), N1-Li1-N2 72.1(1).

The closest synthetic analogy to the reaction producing **4** and **5** is Veith’s report^[11a]^ of the 1,3-diaza-2-silacyclopentene **6 a** as it involves dilithiation of *t*BuN(H)CH_2_CH_2_N(H)*t*Bu (Scheme 2). Distinct from our method, the anticipated dianionic diamide was not isolated but trapped in situ with a dichlorosilane to generate the neutral, as opposed to our anionic, heterocyclopentene. Oddly, **6 a** formed only in highly concentrated solutions,^[11a]^ whereas dilute solutions,^[11b]^ more akin to that employed in our study gave an alternative heterocyclopentane product **6 b**. Veith et al. conceded that the reason for the double hydrogen abstraction from the ethylene backbone in forming **6 a** was unclear. In theory **4** and **5** having potentially labile metal centres primed for participation in salt metathesis reactions could be regarded as intermediates in conversions of this type.

**Scheme 2:**
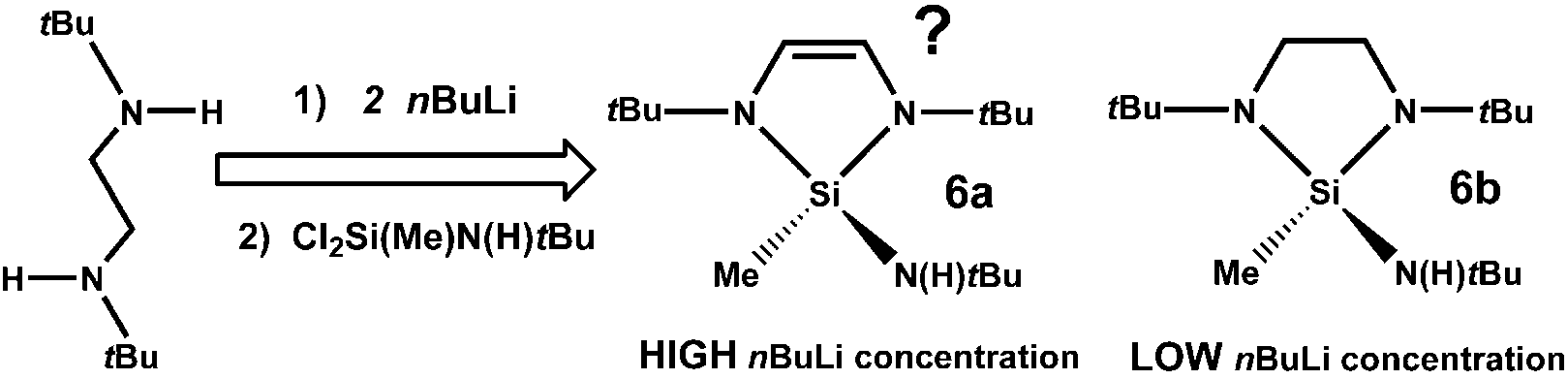
Veith’s reaction of *t*BuN(H)CH_2_CH_2_N(H)*t*Bu with *n*BuLi and further trapping with a dichlorosilane.

A repeat reaction of *t*Bu_2_Zn, *n*BuLi and the diamine in the additional presence of the bulky ketone (*t*Bu)_2_C=O (Scheme 3) may have provided an initial clue to the mechanism behind the formation of **4**. An NMR analysis of the crude reaction revealed a complicated mixture of products,^5^ among which, significantly, **4** and the lithium alkoxide [{*t*Bu_2_C(H)OLi}_4_] (**7)**, were clearly identified. The existence of **7**, the result of a hydride addition to the electrophilic C=O of the ketone, hints at the possible participation of an intermediate hydride species in the N-CH_2_-CH_2_-N to N-CH=CH-N transformation. While the source of the hydride cannot be ascertained with absolute certainty at this juncture, we could straightforwardly rule out that it was coming from either *n*BuLi or *t*Bu_2_Zn,^12^ suggesting that it originates from the ethylene backbone of the diamine. With monoamine dibenzylamine [(PhCH_2_)_2_NH], it has been postulated^13^ that metallation of N-H, followed by β-hydride elimination of “M^+^H^−^” and its subsequent metallation of the remaining NCH_2_ unit (accompanied by H_2_ evolution) accounts for the formation of the 2-aza-allyl anion [{PhC(H)=N-C(H)Ph}^−^]. Similar metallation/β-hydride elimination/re-metallation sequences could be operating in the reaction yielding **4**. For completeness, isolated **7** was also crystallographically characterised. Its molecular structure (see the Supporting Information) is tetrameric with a heterocubane [(μ_3_-OR)_4_Li_4_] core of a type well documented for lithium alkoxides.^14^ These precedents commonly possess additional donor ligands at the Li corners, so **7** belongs to a rarer type^15^ wherein the large bulk of the R group promotes steric saturation of Li at the expense of electronic saturation.

**Scheme 3:**
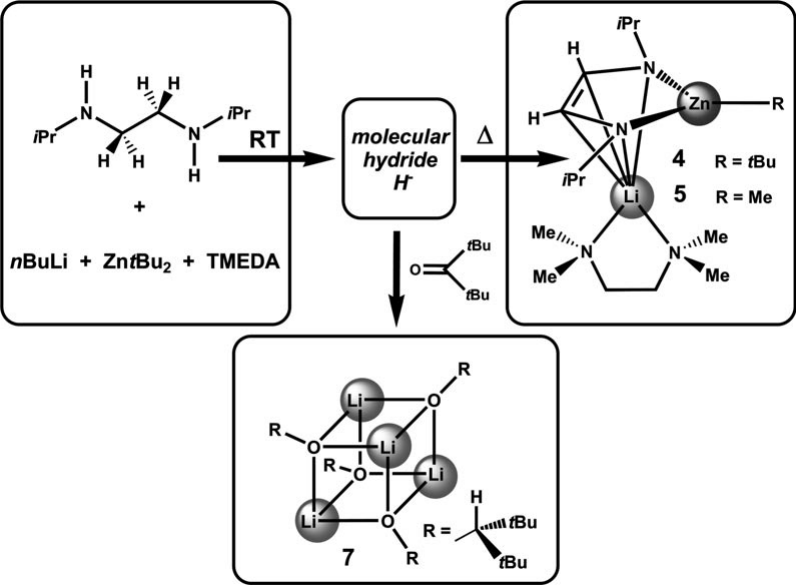
Reaction of *i*PrN(H)CH_2_CH_2_N(H)*i*Pr with a *n*BuLi/R_2_Zn/TMEDA synergic mixture to form 4 or 5 via a putative intermediate hydride which can also be trapped with a ketone to generate a lithium alkoxide.

Noting that the N-CH_2_-CH_2_-N to N-CH=CH-N transformation outlined here for certain will not involve redox processes like that reported by Brookhart et al.^16^ for late transition metal catalysed intramolecular dehydrogenations, in future work we plan to carry out a comprehensive computational study to elucidate the mechanism of this transformation with emphasis on the precise role of the lithium–zinc co-operativity.

## Experimental Section

**General methods**: All reactions and manipulations were carried out in an atmosphere of dry pure argon gas using standard Schlenk and glovebox techniques. *n*-Hexane was distilled from sodium benzophenone. NMR spectra were recorded on a Bruker AVANCE 400 NMR spectrometer, operating at 400.13 MHz for ^1^H, 155.50 MHz for ^7^Li and 100.62 MHz for ^13^C. Data for X-ray crystal structure determination were obtained with a Oxford Diffraction Gemini diffractometer using Mo_Kα_ (*λ* = 0.71073 Å; compounds **5** and **7**) and Cu_Kα_ (*λ* = 1.54180 Å; compounds **1** and **3**) graphite-monochromated radiations. Satisfactory elemental analyses of the compounds could not be obtained due to their high air- and moisture-sensitive nature.

**Synthesis of 1**: A Schlenk tube was charged with of Zn*t*Bu_2_ (4 mmol, 0.72 g), which was dissolved in hexane (20 mL) and one equivalent of *i*Pr(H)NCH_2_CH_2_CH_2_N(H)*i*Pr (4 mmol, 0.72 mL) was added by using a syringe. The resultant colourless solution was allowed to stir overnight at room temperature and heated at reflux temperature for 10 min. To aid crystallisation the solution was concentrated under reduced pressure to a final volume of 2–3 mL and, after standing overnight at −27 °C, colourless crystals of **1** (suitable for X-ray crystallographic analysis) were obtained (0.20 g, 15 %). The low crystalline yield obtained for **1** is just a reflection of its high solubility, being the overall reaction yield almost quantitative as determined by NMR spectroscopic analyses of both **1** and reaction filtrates. ^1^H NMR (400.13 MHz, C_6_D_6_, 293 K): *δ*=2.62 (m, 2 H, CH, *i*Pr), 2.02 (m, 4 H, CH_*2*_), 1.34 (s, 18 H, CH_*3*_, *t*Bu), 0.90 (d, *J*=5.2 Hz, 12 H, CH_*3*_, *i*Pr), 0.85 ppm (s, br, 2 H, NH); ^13^C{^1^H} NMR (100.62 MHz, C_6_D_6_, 293 K): *δ*=49.5 (CH, *i*Pr), 47.2 (CH_2_), 35.8 (CH_3_, *t*Bu), 23.1 (CH_3_, *i*Pr), 19.9 ppm (*C*(CH_3_), *i*Pr).

**Crystallisation of 3**: *n*BuLi (1.25 mL, 2 mmol) was added dropwise to a solution of DPEDA(H_2_) (0.36 mL, 2 mmol) in hexane (10 mL) at 0 °C. This temperature was maintained as TMEDA (0.3 mL, 2 mmol) and a solution of *t*Bu_2_Zn (0.36 g, 2 mmol) in hexane (10 mL) were added giving a pale yellow solution with some white solid. This solution was stored immediately at −27 °C giving a crop of colourless crystals suitable for X-ray crystallographic analysis corresponding to complex **3**. Attempts to characterise the kinetic product **3** by NMR spectroscopy were unsuccessful due to its high thermal instability.

**Synthesis of 4**: *n*BuLi (1.25 mL, 2 mmol) was added dropwise to a solution of DPEDA(H_2_) (0.36 mL, 2 mmol) in hexane (10 mL) at 0 °C. This temperature was maintained as TMEDA (0.3 mL, 2 mmol) and a solution of *t*Bu_2_Zn (0.36 g, 2 mmol) in hexane (10 mL) were added giving a pale yellow solution with some white solid. This solution was refluxed for 2 h producing a bright orange solution. Storing the solution at −70 °C gave a crop of yellow crystals of **4** which were isolated in a 38.9 % (0.30 g) crystalline yield. ^1^H NMR (400.13 MHz, C_6_D_6_, 300 K): δ(ppm)=5.83 (s, 2 H, CH, C*H*=C*H*), 3.48 (m, 2 H, CH, *i*Pr), 1.81 (s, 12 H, CH_3_, TMEDA), 1.60 (s, 9 H, CH_3_, *t*Bu), 1.58 (s, 4 H, CH_2_, TMEDA), 1.40 (d, *J=*6.3 Hz, 6 H, CH_3_, *i*Pr), 1.32 ppm (d, *J*=6.3 Hz, 6 H, CH_3_, *i*Pr); ^13^C{^1^H} NMR (100.62 MHz, C_6_D_6_, 300 K): δ(ppm)=116.3 (CH, CH=CH), 56.0 (CH_2_, TMEDA), 52.7 (CH, *i*Pr), 45.6 (CH_3_, TMEDA), 35.4 (CH_3_, *t*Bu), 28.6 (CH_3_, *i*Pr), 28.0 (CH_3_, *i*Pr), 20.6 ppm (*C*(CH_3_), *t*Bu); ^7^Li (155.50 MHz, C_6_D_6_, 300 K): δ(ppm)= −2.40 ppm.

## Notes

Detailed facts of importance to specialist readers are published as ”Supporting Information”. Such documents are peer-reviewed, but not copy-edited or typeset. They are made available as submitted by the authors.
