# Supplementary material for: Synergic Transformation of an Ethylenediamine to a Lithium 1,3-Diaza-2-zincacyclopentene via an Alkyllithium/Bis(alkyl)zinc Mixture
Source: Chemistry. 2010 Jul 19;16(33):9964–8. doi: 10.1002/chem.201001314 (PMC3784041; doi:10.1002/chem.201001314)

CHEMISTRY
A EUROPEAN JOURNAL

Supporting Information

© Copyright Wiley-VCH Verlag GmbH & Co. KGaA, 69451 Weinheim, 2010

Synergic Transformation of an Ethylenediamine to a Lithium 1,3-Diaza-2-zincacyclopentene via an Alkyllithium/Bis(alkyl)zinc Mixture

Ross Campbell, Pablo García-Álvarez,* Alan R. Kennedy, and Robert E. Mulvey*[a]

chem_201001314_sm_miscellaneous_information.pdf

Supporting Information

Synergic Transformation of an Ethylenediamine to a Lithium 1, 3-Diaza-2-zincacyclopentene via an Alkyllithium/Bis(alkyl)zinc Mixture

Ross Campbell, Pablo García-Álvarez,* Alan R. Kennedy, and Robert E. Mulvey*

WestCHEM, Department of Pure and Applied Chemistry, University of Strathclyde, Glasgow, G1 1XL, U.K.

* To whom correspondence should be addressed.

E-mail: r.e.mulvey@strath.ac.uk

Supporting Information contents

General Methods 3

Crystal data 4-6

Synthesis of [*t*Bu_2Zn·{*i*PrN(H)CH_2CH_2N(H)*i*Pr}] (1) 7

Figures S1-S3 (NMR data of 1) 7-8

Synthesis of [{LiN(*i*Pr)CH_2CH_2N(H)*i*Pr}] 9

Figure S4 (NMR data of [{LiN(*i*Pr)CH_2CH_2N(H)*i*Pr}]) 9

Crystallisation of [*t*Bu_2Zn·{*i*PrN(Li·TMEDA)CH_2CH_2N(H)*i*Pr}]$_3$ (3) 10

Synthesis of [(TMEDA)·Li(*i*PrNCHCHN*i*Pr)Zn(*t*Bu)] (4) 11

Figures S5-S7 (NMR data of 4) 11-12

Synthesis of [(TMEDA)·Li(*i*PrNCHCHN*i*Pr)Zn(Me)] (5) 13

Figures S8-S10 (NMR data of 5) 13-14

Addition of ^{t}Bu_2CO to [{LiN(*i*Pr)CH_2CH_2N(H)*i*Pr}] + TMEDA + Me_2Zn 15

Figures S11-S13 (NMR data of ^{t}Bu_2CO + DPEDA(H)Li + TMEDA + Me_2Zn) 15-16

Isolation of [{*t*Bu_2C(H)OLi}$_4$] (7) 17

Figures S14-S16 (NMR data of 7) 17-18

Figure S17 (Comparison of 7 with ^{t}Bu_2CO + DPEDA(H)Li + TMEDA + Me_2Zn) 19

Reaction of DPEDA(H_2) with 3 equivalents of *n*BuLi 20

Figure S18 (NMR data of DPEDA(H_2) with 3 equivalents of *n*BuLi) 20

Reaction of ^{t}Bu_2CO with BuLi and NMR data (Figures S19-S21) 21-22

General Methods

All reactions and manipulations were carried out in an atmosphere of dry pure argon gas using standard Schlenk and glovebox techniques. *n*-Hexane was distilled from sodium-benzophenone. NMR spectra were recorded on a Bruker AVANCE 400 NMR spectrometer, operating at 400.13 MHz for ^{1}H, 155.50 MHz for ^{7}Li and 100.62 MHz for ^{13}C. Data for X-ray crystal structure determination were obtained with a Oxford Diffraction Gemini diffractometer using Mo-Kα (λ = 0.71073 Å; compounds **5** and **7**) and Cu-Kα (λ = 1.54180 Å; compounds **1** and **3**) graphite monochromated radiations. Satisfactory elemental analyses of the compounds could not be obtained due to their high air- and moisture-sensitive nature. DPEDA(H_2) is *N*, *N*'-diisopropylethylenediamine.

Crystal Data

Crystal Data for **1**: $C_{16}H_{38}N_2Zn$; A colourless crystal with approximate dimensions 0.15 x 0.11 x 0.10 mm gave an orthorhombic space group Pbca, a = 14.1596(17) b = 16.301(2) c = 16.9571(18) Å, V = 3914.0(8) Å^3, T = 123(2) K, Z = 8, ρ_{calc} = 1.099 Mg m^{-3}, $2\theta_{max}$ = 124.96 °, CuK$_\alpha$ λ = 1.54180 Å. R1 = 0.0582 (for 1232 reflections with I>2σ(I)) wR2 = 0.1395 and S = 0.764 for 182 parameters and 3077 unique reflections. Minimum/maximum residual electron density –0.415/0.685 eÅ^{-3}. *Crystal Data* for **3**: $C_{22}H_{53}LiN_4Zn$; A colourless crystal with approximate dimensions 0.10 x 0.08 x 0.04 mm gave a triclinic space group P $\bar{1}$, a = 8.8657(5) b = 10.9105(6) c = 15.2569(9) Å, α = 94.470(5), β = 99.423(5), γ = 103.390(5)°, V = 1405.94(14) Å^3, T = 123(2) K, Z = 2, ρ_{calc} = 1.054 Mg m^{-3}, $2\theta_{max}$ = 146.24 °, CuK$_\alpha$ λ = 1.54180 Å. R1 = 0.0321 (for 4960 reflections with I>2σ(I)) wR2 = 0.0898 and S = 1.061 for 267 parameters and 5545 unique reflections. Minimum/maximum residual electron density –0.266/0.663 eÅ^{-3}. *Crystal Data* for **5**: $C_{15}H_{35}LiN_4Zn$; A colourless crystal with approximate dimensions 0.10 x 0.09 x 0.03 mm gave a monoclinic space group P2$_1$/n, a = 9.7395(5) b = 13.6980(7) c = 15.4433(7) Å, β = 105.833(5)°, V = 1982.15(17) Å^3, T = 123(2) K, Z = 4, ρ_{calc} = 1.152 Mg m^{-3}, $2\theta_{max}$ = 52.00 °, MoK$_\alpha$ λ = 0.71073 Å. R1 = 0.0391 (for 2314 reflections with I>2σ(I)) wR2 = 0.0471 and S = 0.803 for 199 parameters and 3872 unique reflections. Minimum/maximum residual electron density –0.354/0.917 eÅ^{-3}. *Crystal Data* for **7**: $C_{36}H_{76}Li_4O_4$; A colourless crystal with approximate dimensions 0.12 x 0.10 x 0.10 mm gave a monoclinic space group P2$_1$/n, a = 12.3073(4) b = 17.8124(7) c = 18.1275(7) Å, β = 97.765(4)°, V = 3937.5(3) Å^3, T = 123(2) K, Z = 4, ρ_{calc} = 1.013 Mg m^{-3}, $2\theta_{max}$ = 5226.00 °, MoK$_\alpha$ λ = 0.71073 Å. R1 = 0.0493 (for 3615 reflections with I>2σ(I)) wR2 = 0.0881 and S = 0.783 for 421 parameters and 7670 unique reflections. Minimum/maximum residual electron density –0.157/0.195 eÅ^{-3}.

All the structures were solved by direct methods and refined to convergence on F^2 (SHELXL-97; *Acta Cryst*. **2008**, A64, 112.). Crystallographic data (excluding structure factors) for the compounds **1**, **3**, **5** and **7** have been deposited with the Cambridge Crystallographic Data Centre as supplementary publication CCDC 774605, CCDC 774605, CCDC 774605 and CCDC 774605 respectively. Copies of the data can be obtained free of charge on application to CCDC, 12 Union Road, Cambridge CB2 1EZ, UK (fax: (+44) 1223-336-033; email: deposit@ccdc.cam.ac.uk).

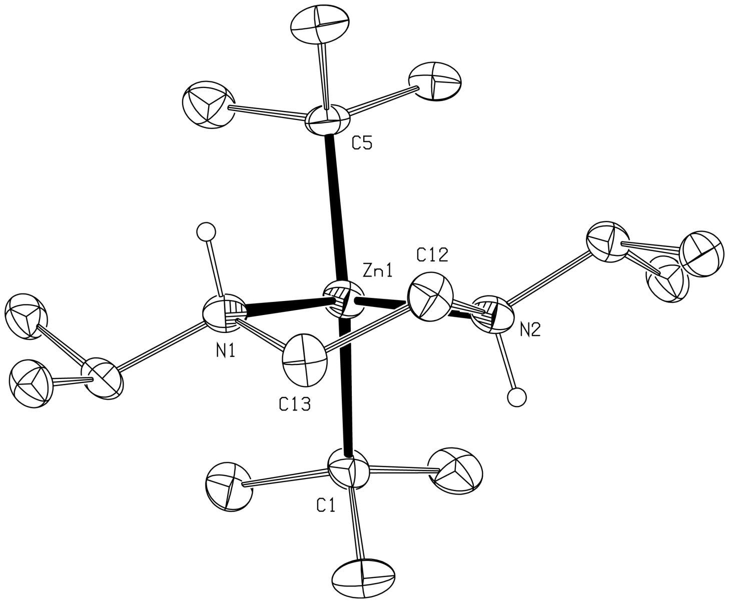

Molecular structure of **1** with hydrogen atoms (except N–*H*) omitted for clarity. Selected bond lengths [Å] and angles [°]: Zn(1)–C(1) 2.063(6), Zn(1)–C(5) 2.042(6), Zn(1)–N(1) 2.244(5), Zn(1)–N(2) 2.255(5), N(1)–C(13) 1.459(7), N(2)–C(12) 1.459(7), C(12)–C(13) 1.512(8), C(5)–Zn(1)–C(1) 133.9(2), C(5)–Zn(1)–N(1) 105.7(2), C(1)–Zn(1)–N(1) 109.7(2), C(5)–Zn(1)–N(2) 110.5(2), C(1)–Zn(1)–N(2) 104.3(2), N(1)–Zn(1)–N(2) 79.6(2).

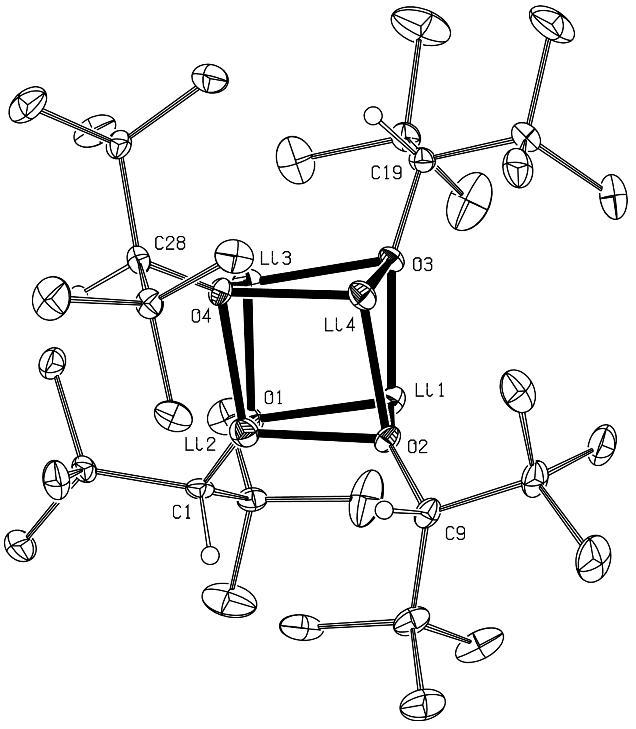

Molecular structure of **7** with hydrogen atoms (except C–*H*) omitted for clarity. Selected bond lengths [Å] and angles [°]: Li(1)-O(3) 1.891(3), Li(1)-O(2) 1.929(3), Li(1)-O(1) 1.929(3), Li(1)-Li(4) 2.473(5), Li(1)-Li(3) 2.475(4), Li(1)-Li(2) 2.487(5), Li(2)-O(2) 1.941(3), Li(2)-O(4) 1.947(3), Li(2)-O(1) 1.948(3), Li(2)-Li(3) 2.446(4), Li(2)-Li(4) 2.458(4), Li(3)-O(1) 1.888(3), Li(3)-O(4) 1.901(3), Li(3)-O(3) 1.941(3), Li(3)-Li(4) 2.479(4), Li(4)-O(4) 1.884(3), Li(4)-O(2) 1.943(3), Li(4)-O(3) 1.946(3), O(1)-C(1) 1.410(2), O(2)-C(10) 1.4225(19), O(3)-C(19) 1.4150(18), O(4)-C(28) 1.412(2), O(3)-Li(1)-O(2) 100.51(16), O(3)-Li(1)-O(1) 98.44(15), O(2)-Li(1)-O(1) 99.71(15), O(2)-Li(2)-O(4) 99.08(15), O(2)-Li(2)-O(1) 98.67(15), O(4)-Li(2)-O(1) 98.49(13), O(1)-Li(3)-O(4) 102.27(14), O(1)-Li(3)-O(3) 98.15(15), O(4)-Li(3)-O(3) 98.22(15), O(4)-Li(4)-O(2) 101.23(15), O(4)-Li(4)-O(3) 98.65(16), O(2)-Li(4)-O(3) 98.12(15), Li(3)-O(1)-Li(1) 80.83(14), Li(3)-O(1)-Li(2) 79.24(13), Li(1)-O(1)-Li(2) 79.79(14), Li(1)-O(2)-Li(2) 79.97(14), Li(1)-O(2)-Li(4) 79.41(14), Li(2)-O(2)-Li(4) 78.53(13), Li(1)-O(3)-Li(3) 80.44(14), Li(1)-O(3)-Li(4) 80.27(14), Li(3)-O(3)-Li(4) 79.25(14), Li(4)-O(4)-Li(3) 81.82(15), Li(4)-O(4)-Li(2) 79.79(14), Li(3)-O(4)-Li(2) 78.92(13).

Synthesis of [*t*Bu$_2$Zn·{*i*PrN(H)CH$_2$CH$_2$N(H)*i*Pr}] (1)

A Schlenk tube was charged with 4 mmol (0.72 g) of Zn^{t}Bu$_2$ which was dissolved in 20 mL of hexane and one equivalent of DPEDA(H$_2$) (4 mmol, 0.72 mL) was added via syringe. The resultant colourless solution was allowed to stir overnight at RT and heated at reflux temperature for 10 min. To aid crystallisation the solution was concentrated under reduced presure to a final volume of 2-3 mL and, after standing overnight –27 °C, colourless crystals of **1** (suitable for X-ray crystallographic analysis) were obtained (0.20 g, 15 %). The low crystalline yield obtained for **1** is just a reflection of its high solubility, being the overall reaction yield almost quantitative as determined by NMR spectroscopic analyses of both **1** and reaction filtrates. ^{1}H NMR (400.13 MHz, C_6D_6, 293 K): δ = 2.62 (m, 2 H, C*H*, *i*Pr), 2.02 (m, 4 H, C*H$_2$*), 1.34 (s, 18 H, C*H$_3$*, *t*Bu), 0.90 (d, *J* = 5,2 Hz, 12 H, C*H$_3$*, *i*Pr), 0.85 (s, br, 2 H, N*H*). ^{13}C{^{1}H} NMR (100.62 MHz, C_6D_6, 293 K): δ = 49.5 (*C*H, *i*Pr), 47.2 (*C*H$_2$), 35.8 (*C*H$_3$, *t*Bu), 23.1 (*C*H$_3$, *i*Pr), 19.9 (*C*(CH$_3$), *i*Pr).

Figure S1. ^{1}H NMR spectrum of **1** obtained in C_6D_6 (300 K).

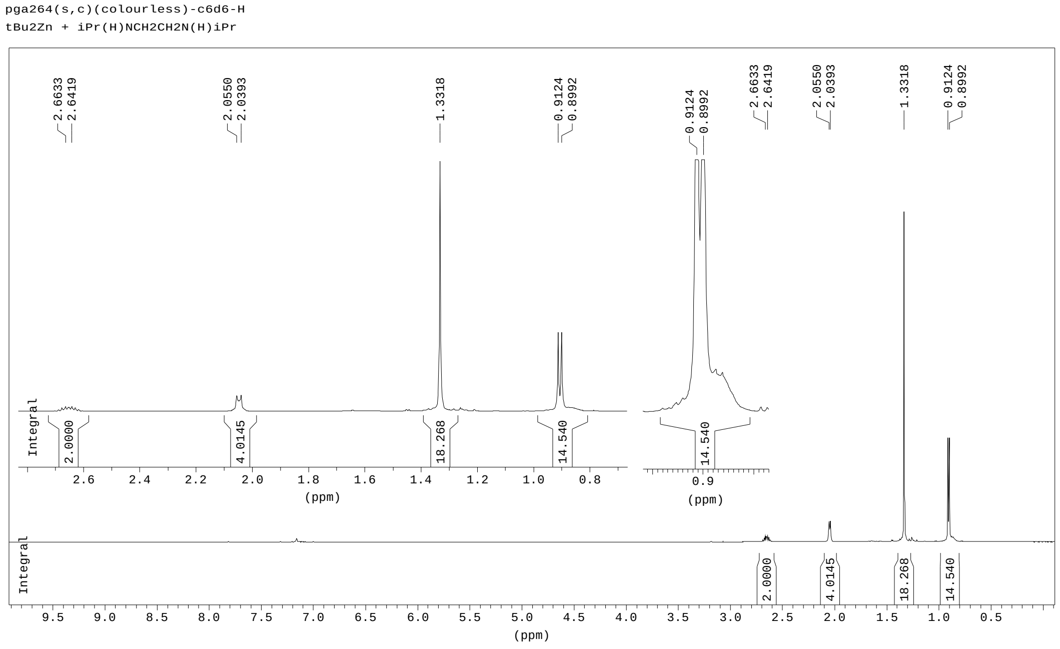

Figure S2. $^{13}C\{^{1}H\}$ NMR spectrum of **1** obtained in C_6D_6 (300 K).

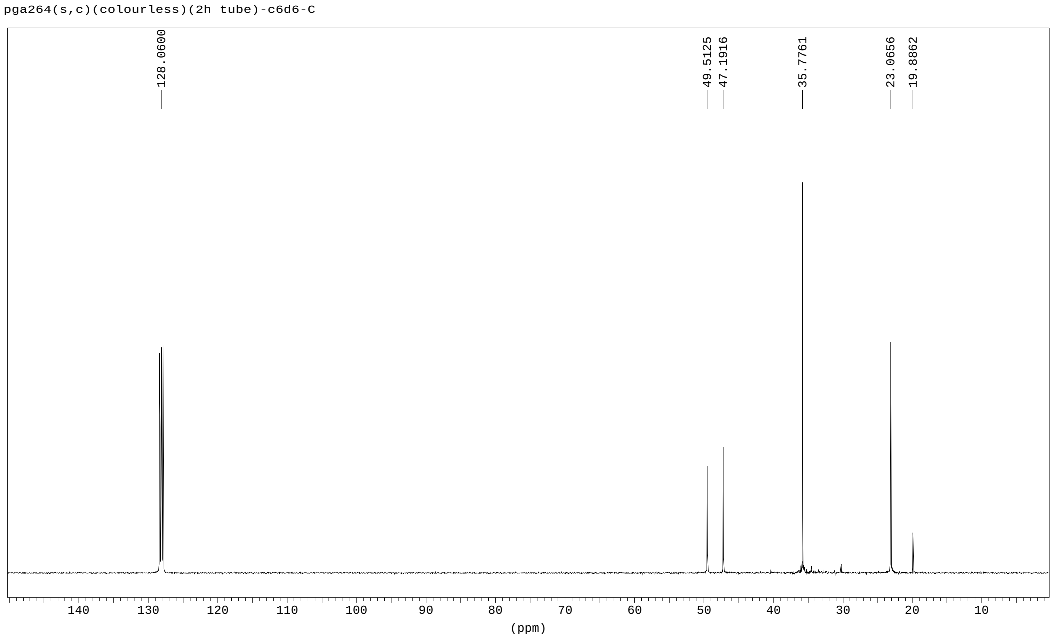

Figure S3. ^{1}H NMR spectrum of reaction filtrate in **1** synthesis in C_6D_6 (300 K).

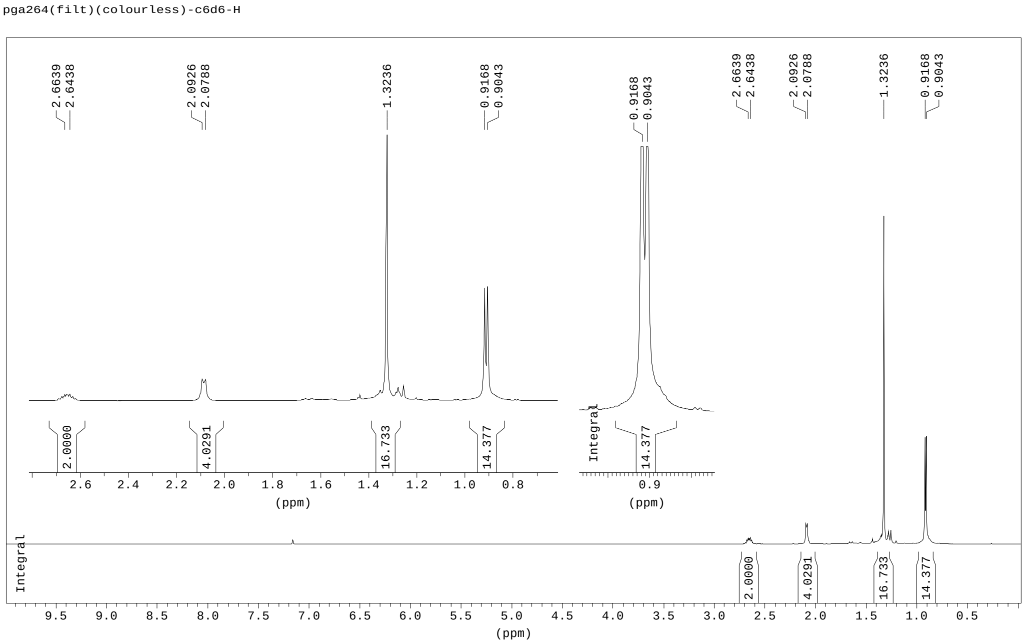

Synthesis of [{LiN(*i*Pr)CH_2CH_2N(H)*i*Pr}]

3.75 mL (6 mmol) BuLi was added dropwise to a solution of 1.08 mL (6 mmol) DPEDA(H_2) in 20 mL of hexane at 0 °C giving a pale yellow solution. This solution was allowed to warm to room temperature and was stirred for 1 h. The solution was concentrated and stored at –30 °C giving a crop of colourless crystals (0.71g, 79 % yield). ^{1}H NMR (400.13 MHz, C_6D_6, 300 K): δ(ppm) = 3.6-2.4 (br, 6 H, C*H* and NCH_2), 1.6-0.7 (br, 12 H, CH_3, *i*Pr), 0.6-0.3 (m, br, 1 H, N*H*). ^{7}Li (155.50 MHz, C_6D_6, 300 K): δ(ppm) = 2.4 (br).

Figure S4. ^{1}H (top) and ^{7}Li (bottom) NMR spectra of [{LiN(*i*Pr)CH_2CH_2N(H)*i*Pr}] in C_6D_6 (300 K).

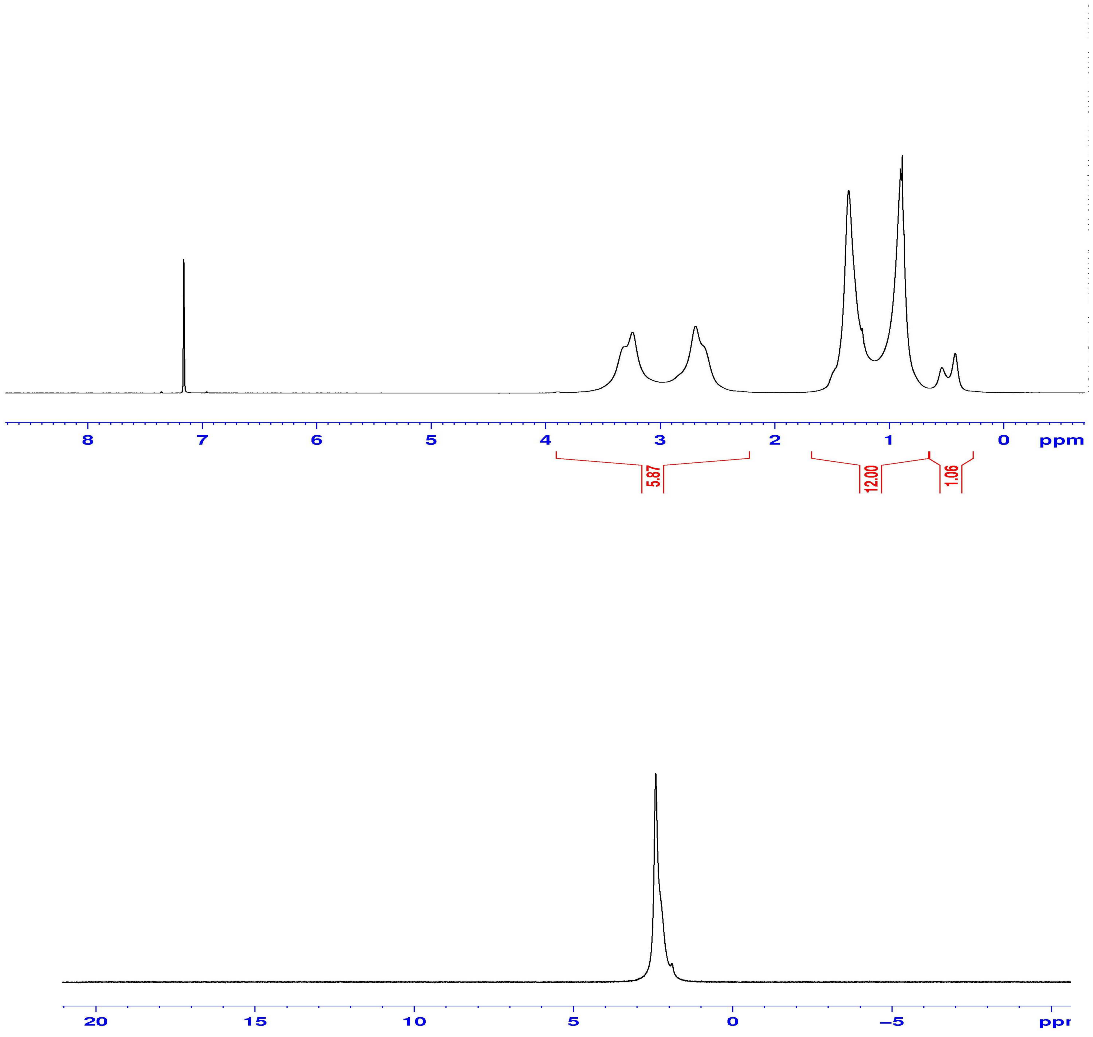

Crystallisation of [tBu$_2$Zn·{iPrN(Li·TMEDA)CH$_2$CH$_2$N(H)iPr}]$_3$ (3)

1.25 mL (2 mmol) *n*BuLi was added dropwise to a solution of 0.36 mL (2 mmol) DPEDA(H$_2$) in 10 mL hexane at 0 °C. This temperature was maintained as 0.3 mL (2 mmol) TMEDA and a solution of 0.36 g (2 mmol) *t*Bu$_2$Zn in 10 mL hexane were added giving a pale yellow solution with some white solid. This solution was stored inmediatly at –27 °C giving a crop of colourless crystals suitable for X-ray crystallographic analysis corresponding to complex **3**. Attempts to characterise by NMR spectroscopy the kinetic product **3** resulted unsuccesful due to its high thermal unstability.

Synthesis of [(TMEDA)·Li(*i*PrNCHCHN*i*Pr)Zn(*t*Bu)] (4)

1.25 mL (2 mmol) *n*BuLi was added dropwise to a solution of 0.36 mL (2 mmol) DPEDA(H_2) in 10 mL hexane at 0 °C. This temperature was maintained as 0.3 mL (2 mmol) TMEDA and a solution of 0.36 g (2 mmol) *t*Bu_2Zn in 10 mL hexane were added giving a pale yellow solution with some white solid. This solution was refluxed for 2 hours producing a bright orange solution. Storing the solution at –70 °C gave a crop of yellow crystals which were isolated in a 38.9 % (0.30g) crystaline yield. ^{1}H NMR (400.13 MHz, C_6D_6, 300 K): δ(ppm) = 5.83 (s, 2 H, C*H*, C*H*=C*H*), 3.48 (m, 2 H, C*H*, *i*Pr), 1.81 (s, 12 H, CH_3, TMEDA), 1.60 (s, 9 H, CH_3, *t*Bu), 1.58 (s, 4 H, CH_2, TMEDA), 1.40 (d, *J* = 6.3 Hz, 6 H, CH_3, *i*Pr), 1.32 (d, *J* = 6.3 Hz, 6 H, CH_3, *i*Pr). ^{13}C{^{1}H} NMR (100.62 MHz, C_6D_6, 300 K): δ(ppm) = 116.3 (*C*H, *C*H=*C*H), 56.0 (*C*H_2, TMEDA), 52.7 (*C*H, *i*Pr), 45.6 (*C*H_3, TMEDA), 35.4 (*C*H_3, *t*Bu), 28.6 (*C*H_3, *i*Pr), 28.0 (*C*H_3, *i*Pr), 20.6 (*C*(CH_3), *t*Bu). ^{7}Li (155.50 MHz, C_6D_6, 300 K): δ(ppm) = –2.40.

Figure S5. ^{1}H NMR spectrum of **4** obtained in C_6D_6 (300 K).

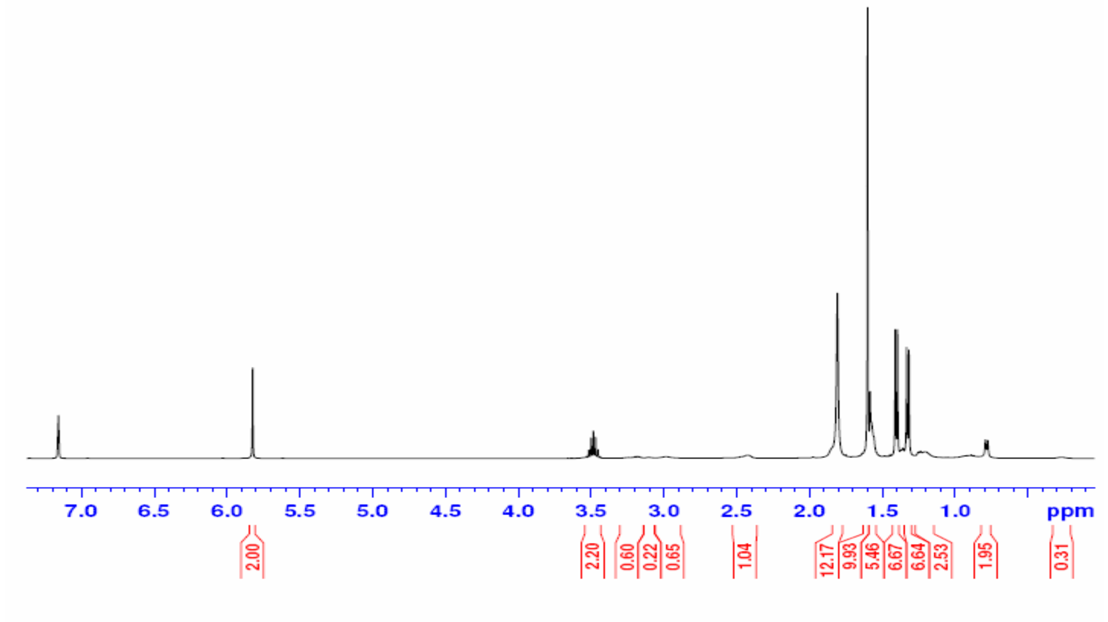

Figure S6. ^{7}Li NMR spectrum of **4** obtained in C_6D_6 (300 K).

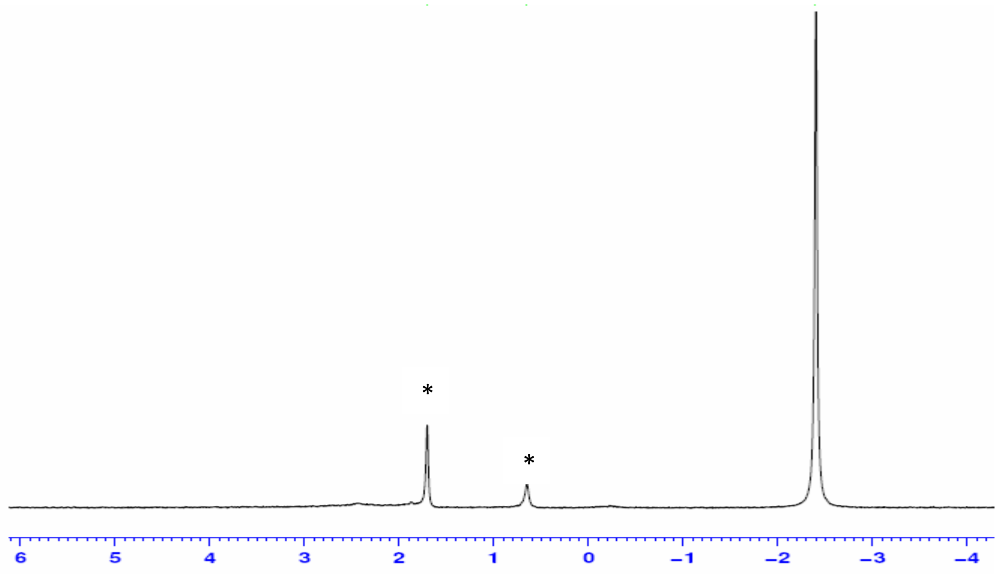

* these small resonances represent intermediates to compound **4** prior to the transformation of the CH_2-CH_2 backbone to CH=CH.

Figure S7. $^{13}C\{^{1}H\}$ NMR spectrum of **4** obtained in C_6D_6 (300 K).

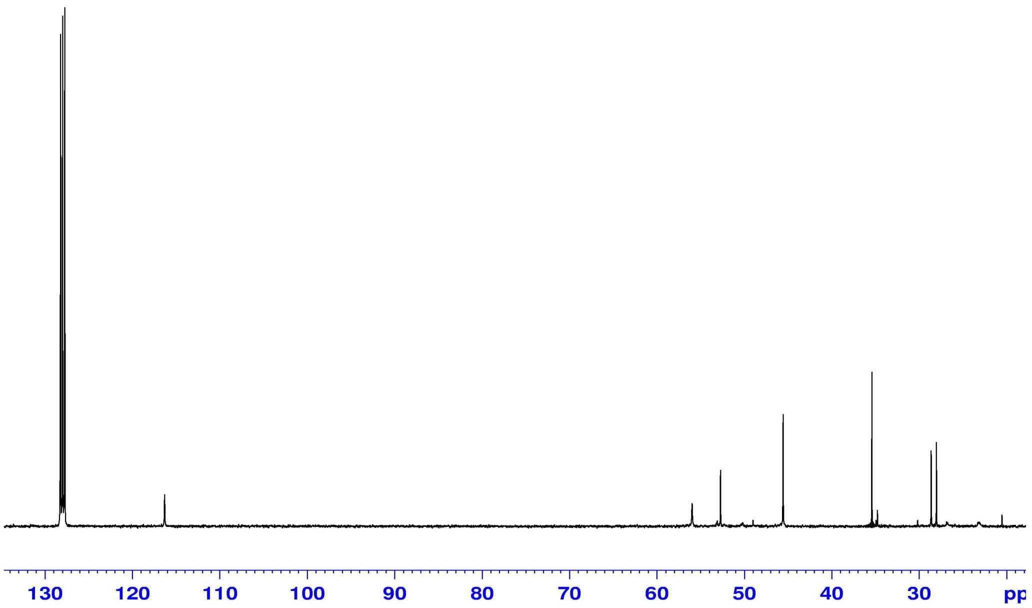

Synthesis of [(TMEDA)·Li(*i*PrNCHCHN*i*Pr)Zn(Me)] (5)

1.25 mL (2 mmol) BuLi was added dropwise to a solution of 0.36 mL (2 mmol) DPEDA(H_2) in 20 mL hexane at 0 °C. This temperature was maintained as 0.3 mL (2 mmol) TMEDA and 2 mL (2mmol) of 1M Me_2Zn in heptane were added giving a pale yellow solution. This solution was refluxed for 1.5 hours producing a bright orange solution. Storing the solution at -30°C gave a crop of yellow crystals (suitable for X-ray crystallographic analysis) which were isolated in a 43.7% (0.30g) crystaline yield. ^{1}H NMR (400.13 MHz, C_6D_6, 300 K): δ(ppm) = 5.93 (s, 2 H, C*H*, C*H*=C*H*), 3.54 (m, 2 H, C*H*, *i*Pr), 1.83 (s, br, 12 H, CH_3, TMEDA), 1.60 (s, br, 4 H, CH_2, TMEDA), 1.42 (d, J = 6.1 Hz, 6 H, CH_3, *i*Pr), 1.30 (d, J = 6.2 Hz, 6 H, CH_3, *i*Pr), –0.02 (s, 3 H, CH_3, ZnCH_3). ^{13}C{^{1}H} NMR (100.62 MHz, C_6D_6, 300 K): δ(ppm) = 116.9 (*C*H, *C*H=*C*H), 56.1 (*C*H_2, TMEDA), 52.5 (*C*H, *i*Pr), 45.6 (*C*H_3, TMEDA), 28.7 (*C*H_3, *i*Pr), 27.7 (*C*H_3, *i*Pr), –11.6 (*C*H_3, Zn*Me*). ^{7}Li (155.50MHz, C_6D_6, 300 K): δ(ppm) = –2.34.

Figure S8. ^{1}H NMR spectrum of **5** obtained in C_6D_6 (300 K).

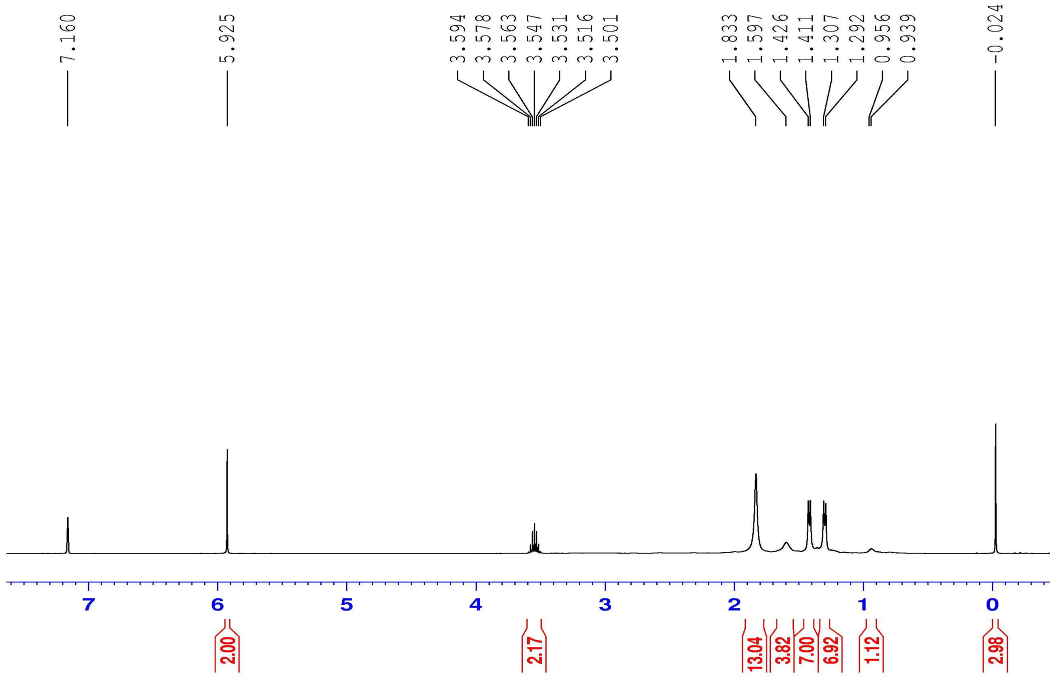

Figure S9. ^{7}Li NMR spectrum of **5** obtained in C_6D_6 (300 K).

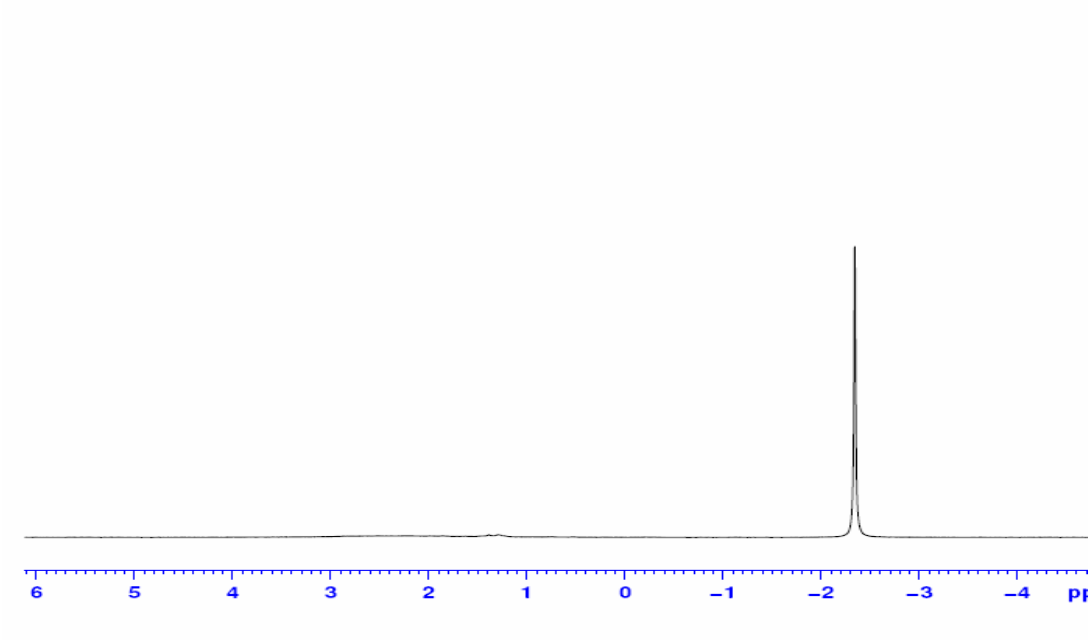

Figure S10. $^{13}C\{^{1}H\}$ NMR spectrum of **5** obtained in C_6D_6 (300 K).

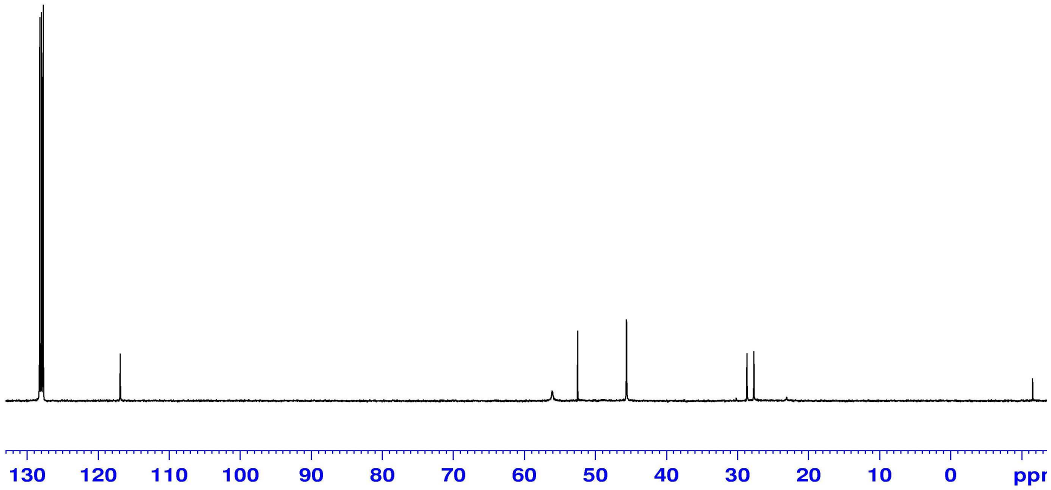

Addition of $^{t}Bu_2CO$ to a mixture of [{LiN(*i*Pr)CH_2CH_2N(H)*i*Pr}], TMEDA and Me_2Zn

1.25 mL (2 mmol) *n*BuLi was added dropwise to a solution of 0.36 mL (2 mmol) DPEDA(H_2) in 20 mL hexane at 0 °C. This temperature was maintained as 0.3 mL (2 mmol) TMEDA and 2 mL (2 mmol) of 1M Me_2Zn in heptane were added giving a pale yellow solution. This solution was allowed to warm to room temperature and stir for 1 h. 0.35 mL (2 mmol) $^{t}Bu_2CO$ was added and the reaction mixture was allowed to stir overnight. An aliquot of the solution was removed, reduced to dryness under vacuum, re-dissolved in C_6D_6 and analysed by NMR spectroscopy. A complicated mixture of products was detected, in which [(TMEDA)·Li(*i*PrNCHCHN*i*Pr)Zn(Me)] (**5**) and the lithium alkoxide [{*t*Bu_2C(H)OLi}$_4$] (**7**) were clearly identified (see Figure S17). Similar results were achieved when replacing Me_2Zn with *t*Bu_2Zn detecting **4** instead of **5**.

Figure S11. ^{1}H NMR spectrum of [{LiN(*i*Pr)CH_2CH_2N(H)*i*Pr}] + Me_2Zn + TMEDA + $^{t}Bu_2CO$ obtained in C_6D_6 (300 K).

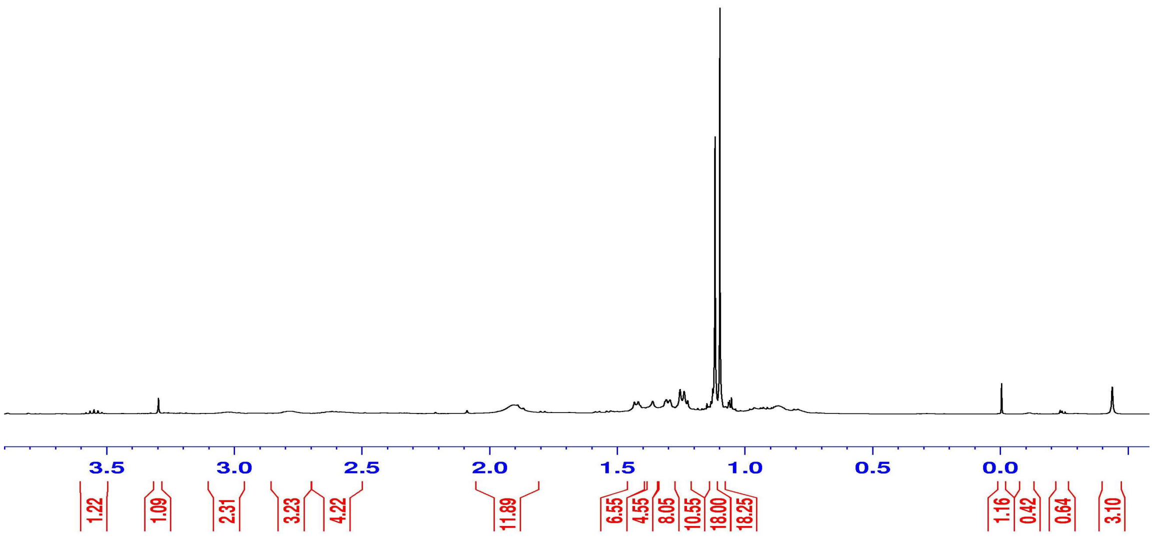

Figure S12. ^{7}Li NMR spectrum of [{LiN(*i*Pr)CH_2CH_2N(H)*i*Pr}] + Me_2Zn + TMEDA + ^{t}Bu_2CO obtained in C_6D_6 (300 K).

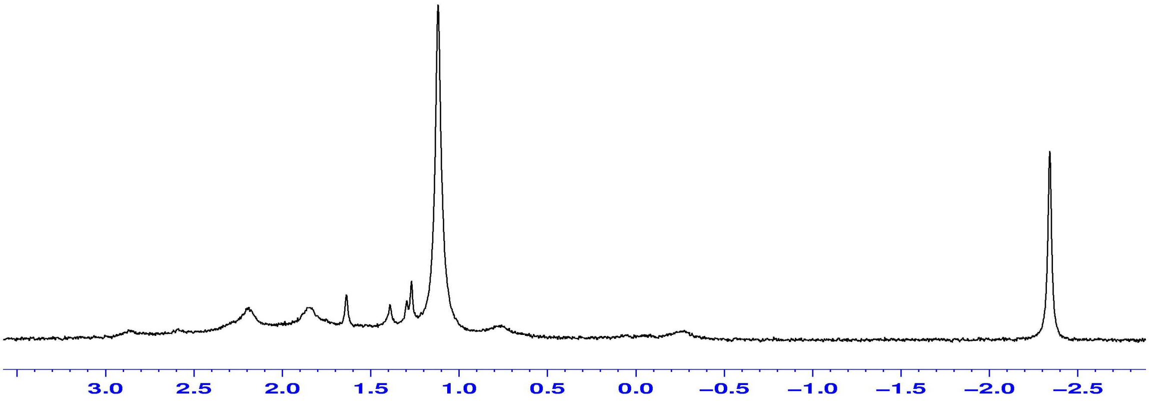

Figure S13. $^{13}C\{^1H\}$ NMR spectrum of [{LiN(*i*Pr)CH_2CH_2N(H)*i*Pr}] + Me_2Zn + TMEDA + ^{t}Bu_2CO obtained in C_6D_6 (300 K).

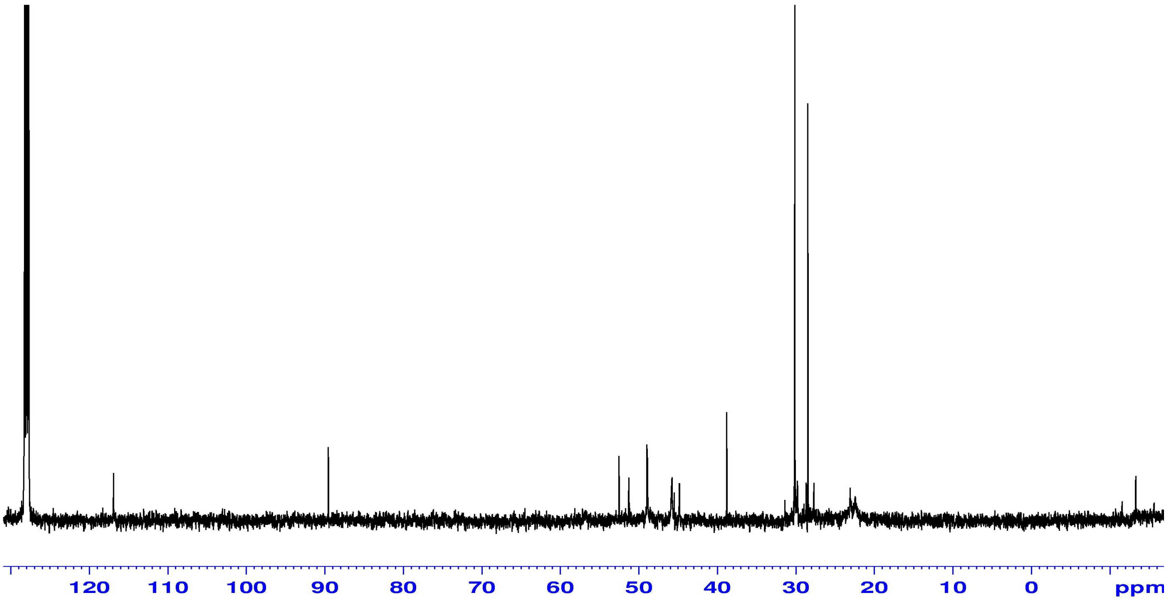

Isolation of [{*t*Bu$_2$C(H)OLi}$_4$] (7)

Repeating the addition of ^{t}Bu$_2$CO to a mixture of [{LiN(*i*Pr)CH$_2$CH$_2$N(H)*i*Pr}], TMEDA and *t*Bu$_2$Zn with an extra equivalent of *n*BuLi facilitated the crystallization (crystals suitable for X-ray crystallographic analysis) of a pure sample of **[{*t*Bu$_2$C(H)OLi}$_4$] (7)** after concentration and storage at –30 ºC. ^{1}H NMR (400.13 MHz, C$_6$D$_6$, 300 K): δ(ppm) = 3.30 (s, 1 H, C*H*), 1.12 (s, 18 H, C*H*$_3$). ^{13}C{^{1}H} NMR (100.62 MHz, C$_6$D$_6$, 300 K): δ(ppm) = 89.6 (*C*H), 38.8 (*C*(CH$_3$)$_3$), 30.1 (*C*H$_3$). ^{7}Li (155.50MHz, C$_6$D$_6$, 300 K): δ(ppm) = 1.12.

Figure S14. ^{1}H NMR spectrum of **7** obtained in C$_6$D$_6$ (300 K).

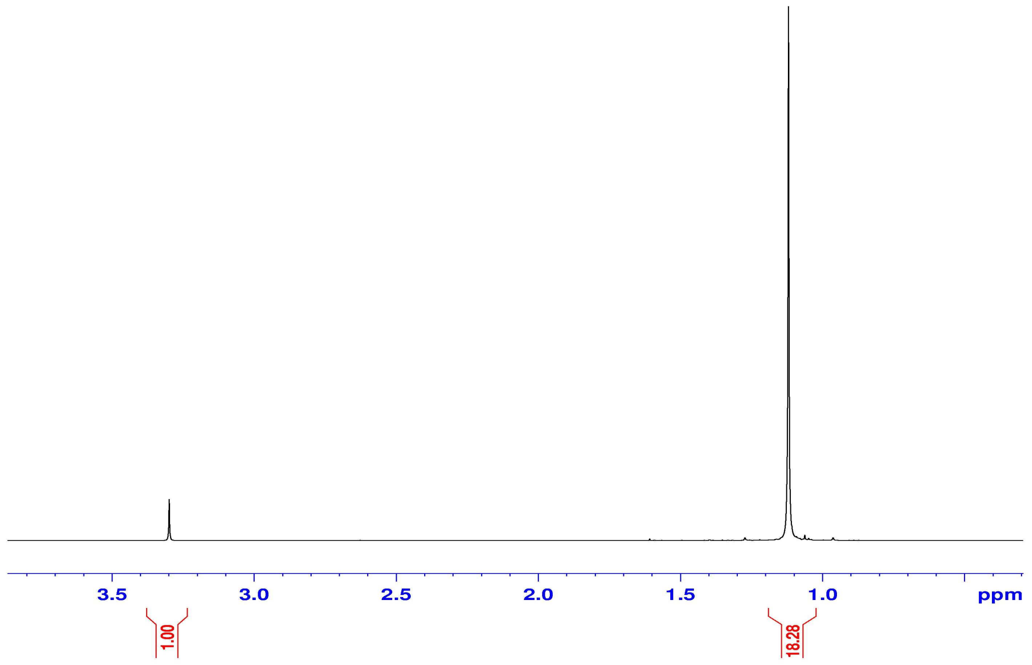

Figure S15. ^{7}Li NMR spectrum of **7** obtained in C_6D_6 (300 K).

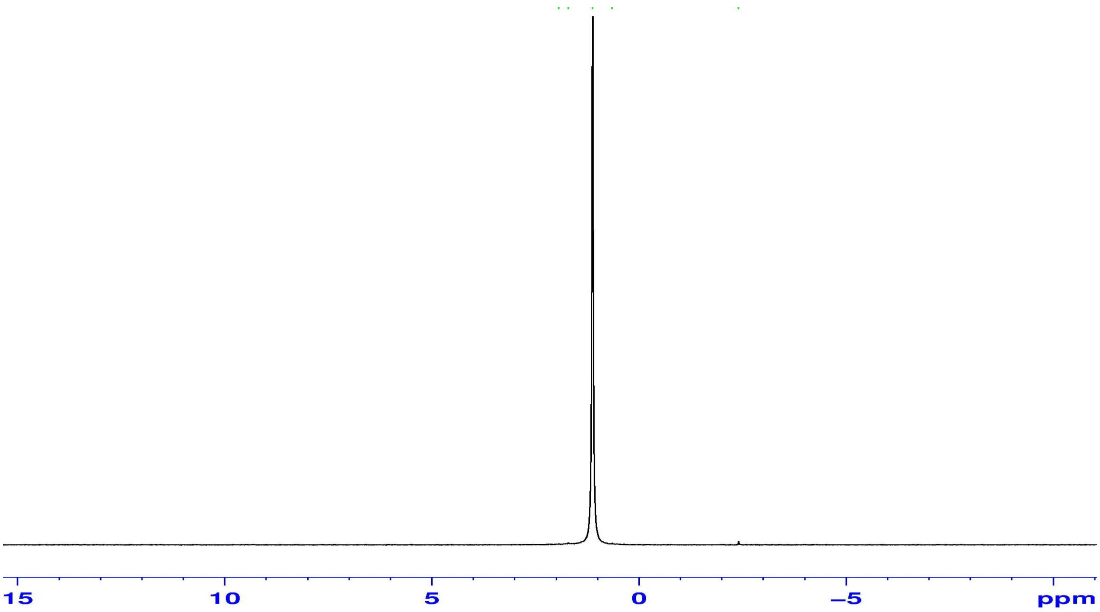

Figure S16. ^{13}C{^{1}H} NMR spectrum of **7** obtained in C_6D_6 (300 K).

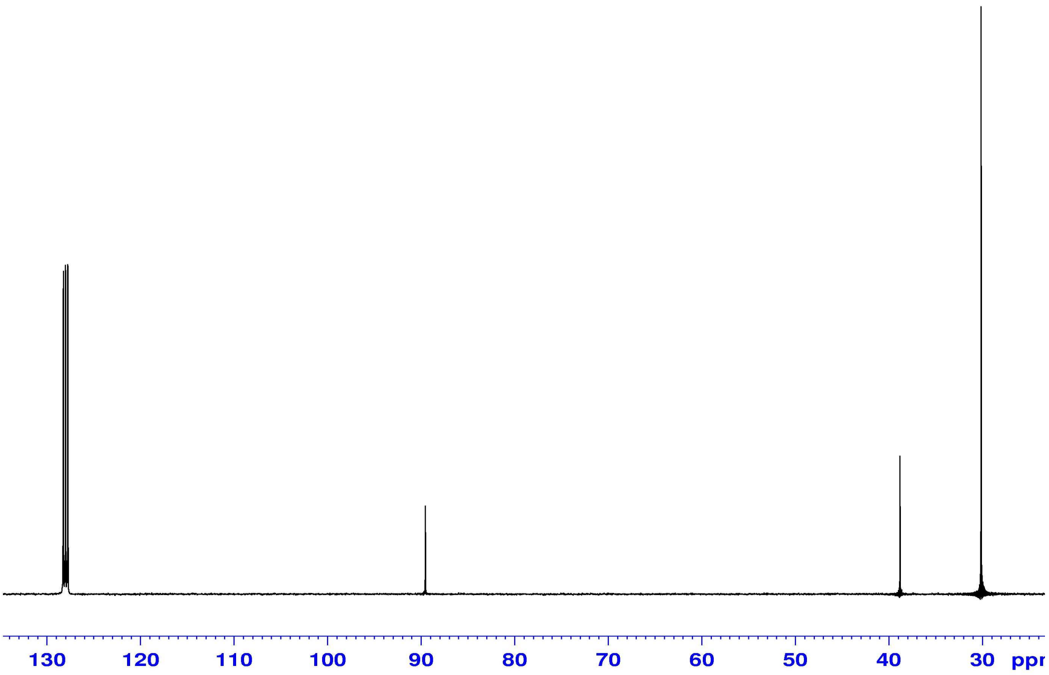

Figure S17. Comparison of the $^{13}C\{^1H\}$ NMR spectrum of **[{*t*Bu$_2$C(H)OLi}$_4$]** (**7**) (top), and [{LiN(*i*Pr)CH$_2$CH$_2$N(H)*i*Pr}] + Me$_2$Zn + TMEDA + ^{t}Bu$_2$CO (bottom), obtained in C$_6$D$_6$ (300 K).

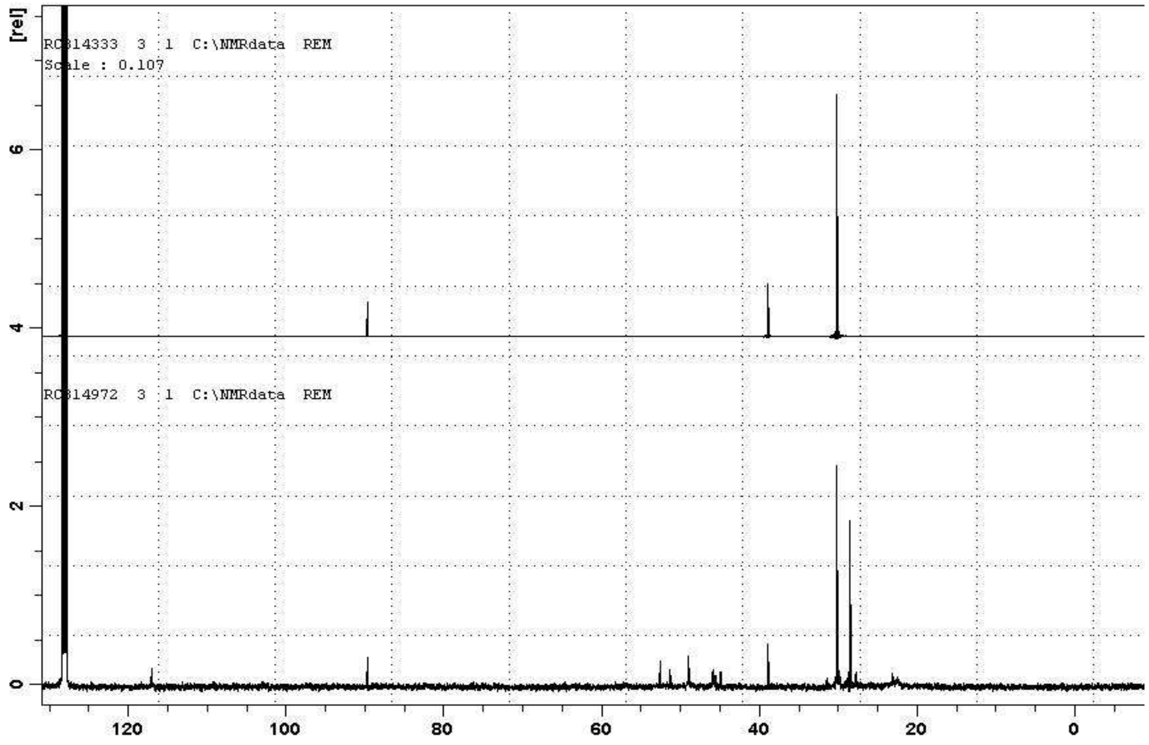

Reaction of DPEDA(H_2) with 3 equivalents of BuLi

3.75 ml (6 mmol) *n*BuLi was added dropwise to a solution of 0.36 mL (2 mmol) DPEDA(H_2) and TMEDA (0.90 mL, 6 mmol) in 20 mL of hexane at 0 °C giving a pale yellow solution. This solution was refluxed for 2 hours (mimicking the reactions conditions in the synthesis of **4** and **5**) giving a dark red solution. An aliquot was removed, reduced to dryness under vacuum, re-dissolved in C_6D_6 and analysed by NMR spectroscopy. The proton NMR spectra revealed just a trace amount of a dehydrogenated species (broad signal about 5.6 ppm) in the reaction mixture. Note that when the reaction was repeated in the absence of TMEDA no significant differences were observed.

Figure S18. ^1H (top) and ^7Li (bottom) NMR spectra of an aliquot of the reaction between 3 equivalents of *n*BuLi and DPEDA(H_2) obtained in C_6D_6 (300 K).

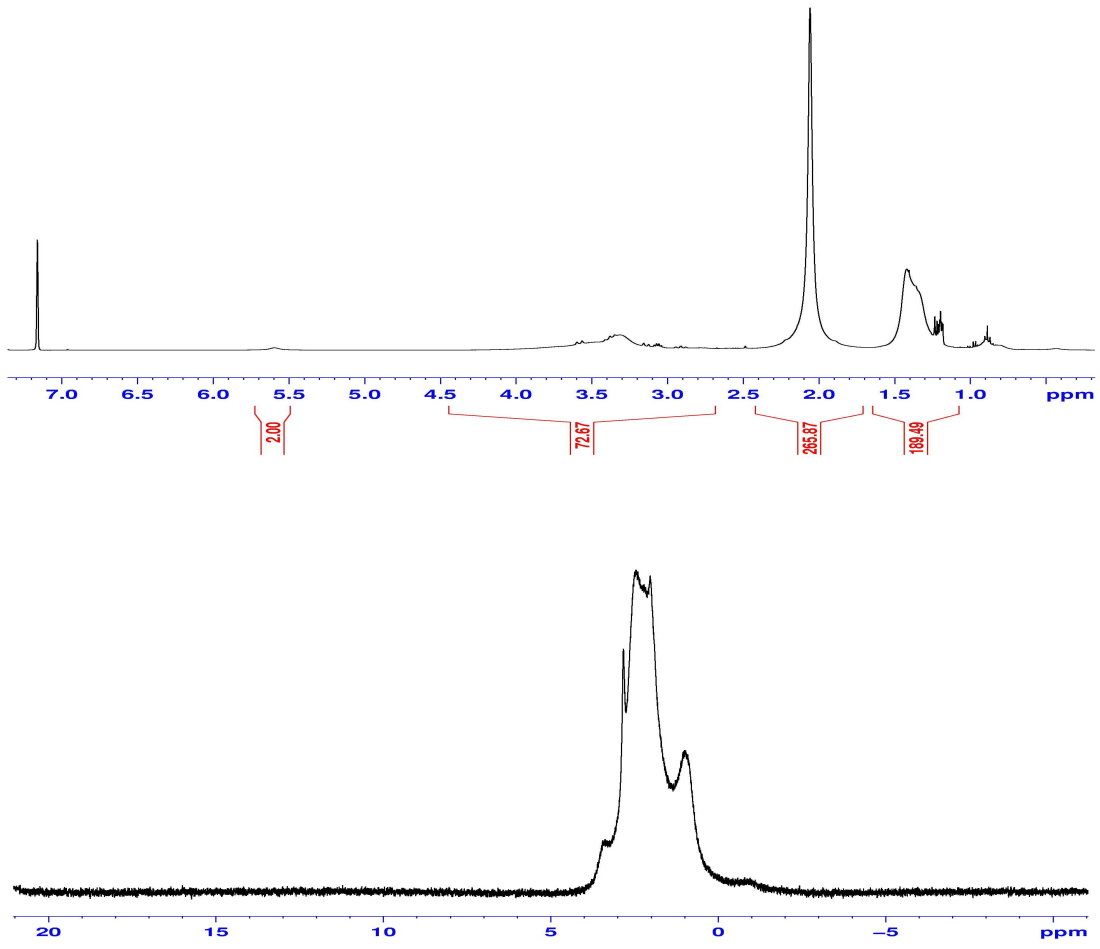

Reaction of ${}^{t}Bu_2CO$ with BuLi

1.25 mL (2 mmol) *n*BuLi was added dropwise to a solution of 0.35 mL (2 mmol) ${}^{t}Bu_2CO$ in 20 mL of hexane. This colourless solution was allowed to stir overnight before an aliquot was removed, reduced to dryness under vacuum, re-dissolved in C_6D_6 and analysed by NMR spectroscopy. This revealed the successful alkylation of the ${}^{t}Bu_2CO$ while no **[{*t*Bu$_2$C(H)OLi}$_4$]** (**7**) was observed.

Figure S19. ^{1}H (top) and ^{7}Li (bottom) NMR spectra of an aliquot of the reaction between *n*BuLi and ${}^{t}Bu_2CO$ obtained in C_6D_6 (300 K).

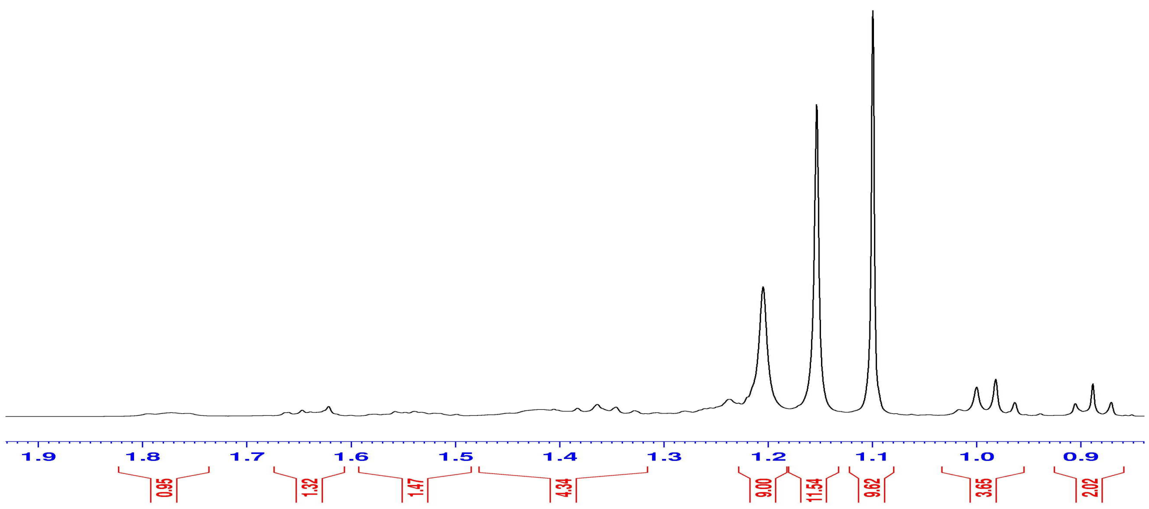

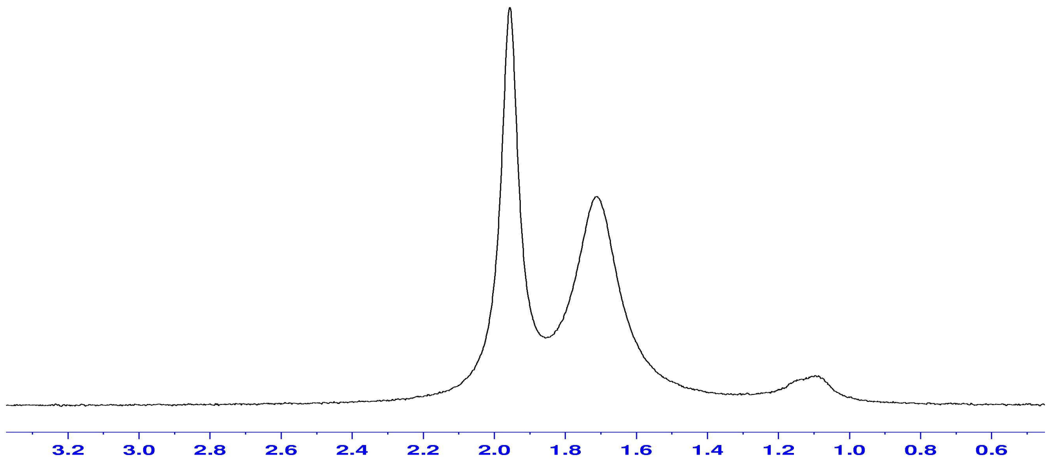

Figure S20. $^{13}C\{^1H\}$ spectrum of an aliquot of the reaction between *n*BuLi and $^tBu_2CO$ obtained in C_6D_6 (300 K).

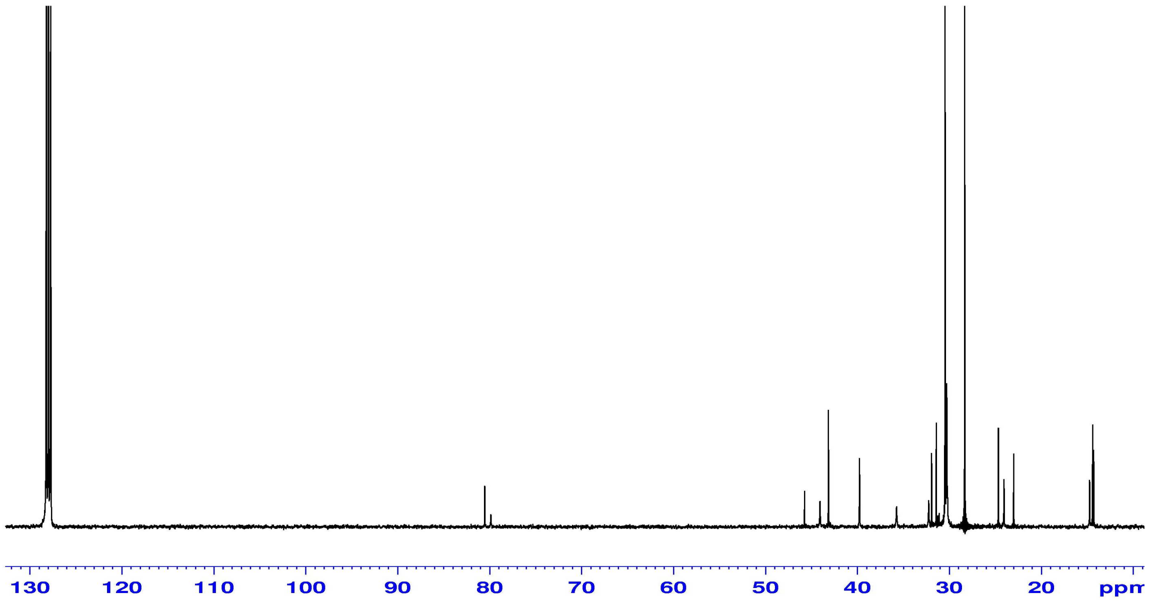

Figure S21. Comparison of the $^{13}C\{^1H\}$ NMR spectra of **[{*t*Bu₂C(H)OLi}₄]** (**7**) (top) and an aliquot of the reaction between *n*BuLi and tBu_2CO (bottom) obtained in C_6D_6 (300 K).

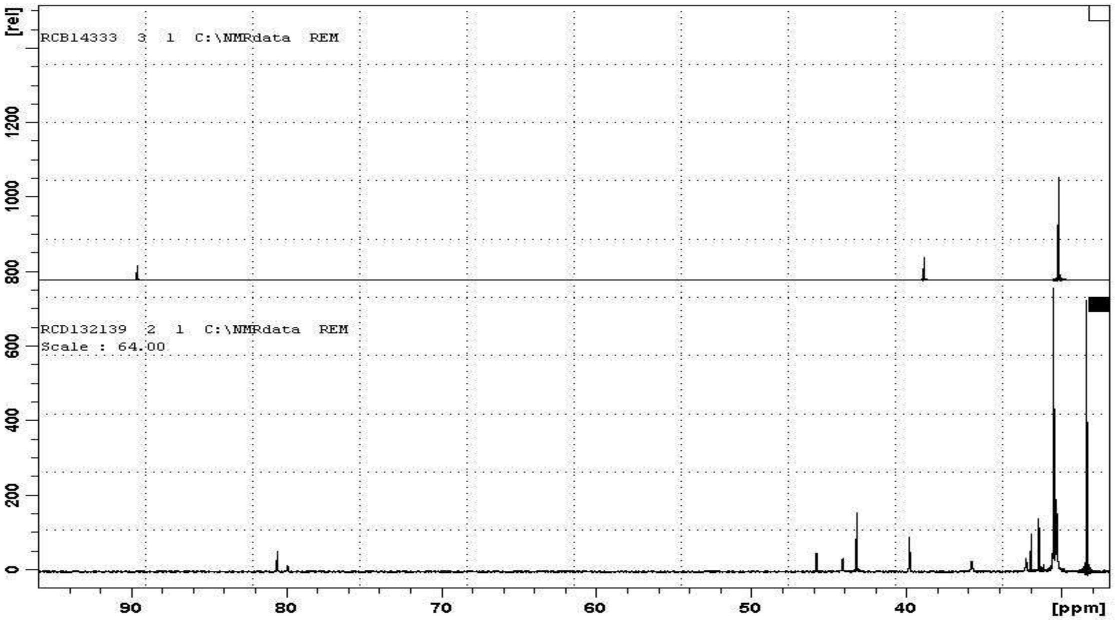

Supplement: Supplementary file 1 [file chem0016-9964-SD1.pdf]
